# The Influence of Polyols on the Process Kinetics and Bioactive Substance Content in Osmotic Dehydrated Organic Strawberries

**DOI:** 10.3390/molecules27041376

**Published:** 2022-02-17

**Authors:** Artur Wiktor, Magdalena Chadzynska, Katarzyna Rybak, Magdalena Dadan, Dorota Witrowa-Rajchert, Malgorzata Nowacka

**Affiliations:** Department of Food Engineering and Process Management, Institute of Food Sciences, Warsaw University of Life Sciences—SGGW, 02-787 Warsaw, Poland; artur_wiktor@sggw.edu.pl (A.W.); mchadzynska1@gmail.com (M.C.); katarzyna_rybak@sggw.edu.pl (K.R.); magdalena_dadan@sggw.edu.pl (M.D.); dorota_witrowa_rajchert@sggw.edu.pl (D.W.-R.)

**Keywords:** strawberry, osmotic dehydration, polyols, polyphenols, antioxidants, vitamin C, polyphenols, anthocyanins, sugar profile, colour, structure

## Abstract

In recent years, an increasing interest in reducing sugar consumption has been observed and many studies are conducted on the use of polyols in the osmotic dehydration process to obtain candied or dried fruits. The studies in the literature have focused on the kinetics of the process as well as the basic physical properties. In the scientific literature, there is a lack of investigation of the influence of such polyol solutions such as sorbitol and mannitol used as osmotic substances during the osmotic dehydration process on the contents of bioactive components, including natural colourants. Thus, the aim of the study was to evaluate the impact of polyols (mannitol and sorbitol) in different concentrations on the process kinetics and on chosen physical (colour and structural changes) as well as chemical (sugars and polyol content, total anthocyanin content, total polyphenol content, vitamin C, antioxidant activity) properties of osmotic-dehydrated organic strawberries. Generally, the results showed that the best solution for osmotic dehydration is 30% or 40% sorbitol solutions, while mannitol solution is not recommended due to difficulties with preparing a high-concentration solution and its crystallization in the tissue. In the case of sorbitol, the changes of bioactive compounds, as well as colour change, were similar to the sucrose solution. However, the profile of the sugar changed significantly, in which sucrose, glucose, and fructose were reduced in organic strawberries and were partially replaced by polyols.

## 1. Introduction

Strawberries (*Fragaria × ananassa*) are valued for their excellent taste, characteristic aroma, and bright red colour [1]. The fruits have been gaining popularity in recent years. The global production of strawberries has risen over 50% between 1999 and 2019 [2]. Strawberries are considered as a great source of vitamins and minerals, particularly vitamin C, folate, manganese, and potassium. They are also rich in polyphenols known for their antioxidant and anti-inflammatory properties [3]. However, strawberry harvest is seasonal, and the fruits are perishable, susceptible to mechanical damage, and easily spoiled. For this reason, technological operations are used to preserve the fruits [4]. Furthermore, in recent years, due to environmental pollution and greater interest in a healthy diet, there has been a growing interest in organic fruit and vegetables [5].

Microbial and chemical stability of foods is affected by the thermodynamic state of water expressed by water activity, which can be lowered either by the addition of solutes or by the removal of water. Osmotic dehydration (OD) allows for both ways to be used at the same time [6]. It is performed by immersion of plant tissue in a hypertonic solution. During OD, a counter-current transfer of mass occurs: water flows from the food material to the osmotic solution, and simultaneously, the osmotic agent goes from the solution into the food. Kinetics of the process depend on osmotic pressure, which is affected by many factors, including the type of osmotic solute [7,8,9]. The molecular weight must be taken into account when choosing the osmotic agent. A low-molecular-weight osmotic substance produces high osmotic pressure and can more easily penetrate the food tissue in comparison to a high-molecular-weight osmotic substance [10,11]. Furthermore, solubility, cost, convenience, non-toxicity, additional preservation effect and compatibility with the dehydrated material must be considered in selecting the osmotic agent [8,12]. The most popular osmotic agent for fruit is sucrose [13,14,15]. However, the current trends in OD include the application of alternative osmotic substances [16]. One trend is the use of ingredients with high nutritional value, for example, fruit juice concentrates [17,18,19], while the other is the use of ingredients with a low glycaemic index such as polyols [20,21,22]. This issue is also important due to the problem of obesity and overweight related to high sugar consumption and its impact on human health [23].

Polyols, also called sugar alcohols or polyhydric alcohols, are a group of sweeteners that are characterized by strong dehydrating capabilities and sweet taste, in which the strength varies from 0.3 to 1 as compared with sucrose and low caloric content [24,25]. Moreover, they exhibit anti-caries, prebiotic, antioxidant, and antibacterial effects and do not affect glucose levels in the blood [26]. Products containing polyols can have the following health claims on the label: ‘do not promote tooth decay’ and ‘reduced speed of digestion and absorption results in lower glycaemic response’ [27]. However, although acceptable daily intake (ADI) has not been specified for sugar alcohols, laxative effects may occur in case of their excessive consumption due to incomplete and delayed absorption [25].

There are seven polyols which are defined as nutritive sweeteners according to the European Union legislation, i.e., sorbitol, mannitol, isomalt, maltitol, lactitol, xylitol and erythritol [28]. Recently, researchers have focused on the use of erythritol [22,29,30,31,32], xylitol [21,22,24,31,32] and maltitol [21,22,24,31,32] as osmotic agents, whereas there are few studies concerning the application of sorbitol [33,34], mannitol [35], isomalt [32] and lactitol in the OD process. Sorbitol and mannitol are six-carbon polyols that have the same molecular weight of about 182 and differ only in the position of the hydroxyl group on carbon 2 in the molecule. Despite the similar structure, they vary in application and properties [36]. Sorbitol can be used as a humectant, softener, and texturizing and anti-crystallizing agent, while mannitol has found to have applications as an anticaking and bulking agent, humectant, stabilizer, sweetener, and thickener. Sorbitol is a white, crystalline, hygroscopic, well-soluble powder, and mannitol is a white, crystalline, non-hygroscopic substance that is one of the least-soluble polyhydric alcohols [26].

The use of polyol solutions can accelerate mass exchange, reduce water activity [31], and diffuse sugars [22] compared to sucrose solution. Furthermore, the OD process in the sugar alcohol solutions conducted before drying might positively affect the drying time and retention of bioactive compounds in the dehydrated material [24]; it may also result in lower caloric value, the safe level of polyols in the final product, and a cooling effect felt in the mouth [30]. To the best of our knowledge, in the scientific literature, there is a lack of investigation on fruits and vegetables and on the influence of such polyol solutions such as sorbitol and mannitol being used as osmotic substances during the OD process on the contents of bioactive components, including natural colourants.

Polyphenols are one of the most important antioxidants in plants. This group of water-soluble compounds includes both phenolics acids and flavonoids (with anthocyanins). Due to their protection against civilization diseases, the maintenance of high content of polyphenols is important [37]. Anthocyanins are a group of water-soluble flavonoids, which show many biological activities in plants, such as imparting stress resistance, extending fruit life or attracting pollinating insects [38]. Moreover, anthocyanins supplied with food have a beneficial effect on the human body through preventing the accumulation of cholesterol in blood vessel walls, preventing cardiovascular diseases and cancers or by regulating blood sugar concentration [39]. Anthocyanins are dominant pigments in strawberry; thus, they are responsible for the red colour of this fruit. Glycosylation increases anthocyanin stability and thus the redness of strawberries, which is favourable by consumers [38,40]. Vitamin C is a water-soluble vitamin that can be lost from a food product through oxidation and/or direct leaching during both processing and storage [41]. It is a sensitive component of food, and its losses can be evaluated regarding the quality of the product after processing [42]. Strawberries are an important source of this vitamin for human nutrition. The content of vitamin C in these fruits equals 58.8 mg/100 g [1]; thus, consumption of about 140 g of strawberries covers the need for this compound, which is 80 mg [43].

Thus, the aim of this study was to evaluate the influence of polyols as an osmotic agent on the process kinetics as well as chosen physical and chemical properties of osmotic-dehydrated organic strawberries. To assess the bioactive activity, a study of total anthocyanin content (TAC), total polyphenol content (TPC), and vitamin C as well as antioxidant activity with DPPH, ABTS radicals, and reducing power (RP) of organic strawberries subjected to osmotic dehydration were evaluated. Furthermore, the colour and structural changes were assessed. In addition, after the osmotic dehydration process, sugar and polyol contents in organic strawberries were studied.

## 2. Results and Discussion

### 2.1. Effect of Different Concentrations of Polyols on Osmotic Dehydration Process of Organic Strawberries

**Figure 1:**
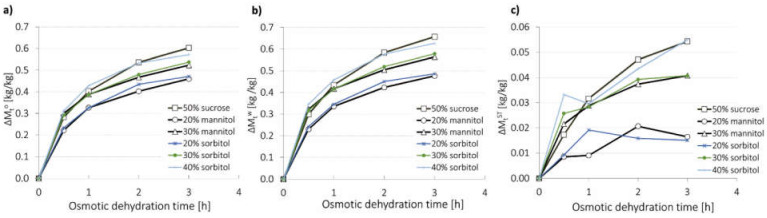
Kinetics of osmotic dehydration of organic strawberries in sucrose, mannitol and sorbitol solutions: (**a**) total mass loss (Mt°), (**b**) water mass loss (Mtw) and (**c**) solids gain (MtST).

The total mass of the sample decreased during OD (Figure 1a) due to the fact that water flow from the plant tissue was considerably bigger than the solute flow into the food material. The rate of total mass loss was the highest at the beginning of the process. This may be explained by decreasing the osmotic pressure difference between the food and the solution over the processing time. According to Tiroutchelvame et al. [44], in order to avoid a considerable reduction in the total mass loss rate over the processing time due to a significant dilution of the osmotic solution, a high amount of the solution-to-sample ratio must be chosen. Researchers observed that increasing the sample-to-solution mass ratio from 1:5 to 1:13 caused a major increase in total mass loss. However, beyond the 1:13 ratio, mass transfer properties during OD had decreased and the operational cost of the process increased. Akbarian et al. [8] suggested that when investigators aim to monitor mass exchange, they usually use a lower solution-to-sample ratio (3:1 or 4:1). Such a claim was confirmed in different studies [18,29,31]. Furthermore, the concentration of the osmotic agent influences total mass loss. In the case of the use of 20%, 30% and 40% sorbitol solutions, total mass loss after 3 h equalled 0.47, 0.54, and 0.57 kg/kg, respectively, while in the case of the use of 20% and 30% mannitol solutions, total mass loss after 3 h equalled 0.46 and 0.52 kg/kg, respectively. Higher total mass loss is a consequence of an increase in osmotic pressure gradient due to a higher concentration of the solute. Similar results concerning the relationship between the solution concentration and total mass loss were obtained by Brochier et al. [34] and Tiroutchelvame et al. [44].

Water mass loss increased with the time of the process; however, the rate of the growth decreased. It was most dynamic during the first 0.5 h of the process in all cases, and depending on the type of solution, this parameter reached from 45.6% to 57.5% after this time in relation to the total water mass loss observed at the end of OD (Figure 1b). A decrease in rate of water mass loss is caused by changes in the tissue structure as well as decreasing osmotic pressure difference between the dehydrated tissue and the surrounding solution [45]. The greatest intensity of water mass loss at the beginning of the process was also reported by Brochier et al. [46] and Mendonça et al. [32] during OD of yacon in glycerol and sorbitol solutions and xylitol, maltitol, erythritol, isomalt and sorbitol solutions, separately. A greater concentration of the osmotic substance resulted in an increase in gradient of osmotic pressure between the plant material and the solution and an increase in water mass loss for both polyols was observed. Similar results concerning the impact of the osmotic agent concentration on water mass loss were noted when apples were osmo-dehydrated in erythrytol, xylitol and maltitol solutions [11]. Based on the water mass loss, it can be stated that OD in sorbitol and mannitol solutions at the same concentrations is characterized by similar efficiency. Differences noted after 3 h of the process between water mass loss from samples dehydrated in sorbitol and mannitol solutions at both 20% and 30% concentrations were not significant (Table 1). It may be explained by the fact that those polyols have the same molecular weight which affects the osmotic pressure during OD. Additionally, no significant differences were observed after 3 h of OD when 40% sorbitol and 50% sucrose solutions were used. This is due to lower molecular weight of sorbitol (182.18 g/mol) compared to sucrose (342.3 g/mol). Because of mannitol crystallization (see Section 2.5), 40% solution of this polyol was not applied. This phenomenon may limit the use of mannitol in the OD process. Moreover, sorbitol is better tolerated and sweeter than mannitol [36]. These properties indicate that sorbitol seems to have wider use as an osmotic substance than mannitol.

Solids gain affects mass exchange during OD to a lesser extent than water mass loss [11]. However, it has great impact on the properties of the final product. When polyol solutions were used, water mass loss reached values in the range of 0.48 to 0.63 kg H_2_O/kg (Figure 1b), whereas solids gain was between 0.02 and 0.05 kg d.m./kg (Figure 1c). Considerably lower solids gain than water mass loss was also reported by Assis et al. [33] during OD of apples in sucrose and sorbitol solutions and by Dermesonlouoglou and Giannakourou [47] during OD of apricot multi-component solutions containing glycerol, erythritol, sodium chloride, calcium chloride, steviol glucoside, and Citrox©. A prolongation of OD caused an increase in solids gain when 30% mannitol, 30% and 40% sorbitol, and 50% sucrose solutions were applied. However, the penetration of the osmotic substance into the tissue was the most intense during the first 0.5 h of OD. Solids gain increases with the growth of osmotic agent concentration and decreases with the growth of its molecular weight. For this reason, significant differences between samples dehydrated in mannitol and sorbitol solutions at the same concentrations were not reported (Table 1). Furthermore, OD in sucrose solution at higher concentration and bigger molecular weight was characterized by solids gain that did not differ significantly from the values of solids gain obtained for the samples osmo-dehydrated in sorbitol solutions of lower concentrations (30% and 40%) and lower molecular weight as well as in mannitol solution of lower concentration (30%) and lower molecular weight.

### 2.2. Effect of Different Concentrations of Polyols on Colour Parameters of Organic Strawberries

The strawberries are characterized by the L*, a*, and b* parameters as 34.3, 25.8, and 21.4, respectively. The osmotic dehydration process resulted in colour changes (Table 2) which are confirmed by other studies. For example, apricot dehydrated in sorbitol solution was characterized by lower L* values and higher a* and b* values which might be related to used osmotic dehydration solutions and the shrinkage effect [47], while in apples dehydrated in erythritol, xylitol, and maltitol solutions [22], the lightening in erythritol as well as for xylitol and maltitol decreased in L* value during osmotic dehydration were noticed.

**Figure 2:**
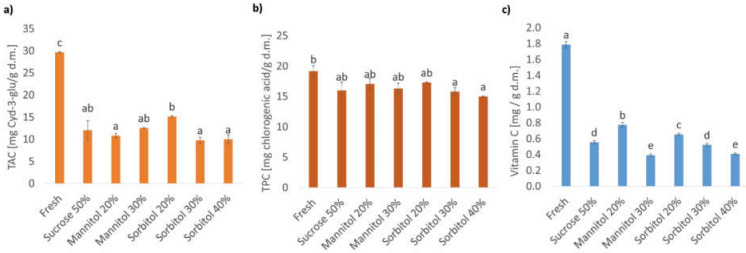
Bioactive compounds in organic strawberries after 3 h of osmotic dehydration in sucrose, mannitol, and sorbitol solutions: (**a**) total anthocyanin content (TAC); (**b**) total phenolics content; (**c**) vitamin C content. a–e—different letters above columns indicate statistically significant differences between the samples by the Tukey test (*p* < 0.05).

L* parameter, responsible for brightness, after osmotic dehydration was significantly higher, which means that the osmo-dehydrated samples were brighter. The highest changes were noticed for samples dehydrated in sucrose, where the L* parameter increased from 34.3 for fresh to 43.6 for osmotic dehydrated in 50% sucrose solution. The polyols used as osmotic agents resulted, in most cases, significantly smaller changes compared to sucrose solution. However, there were not significant changes between samples treated with different polyols. Furthermore, L* parameter was significantly correlated with total mass loss (M_t_°) and water mass loss (M_t_^w^) (M_t_°: r = 0.828, M_t_^w^: r = 0.825). This shows that some colourants can be lost during the osmotic dehydration process, which results in a brighter colour of the strawberry tissue. However, despite the significant degradation of anthocyanins after the OD in each osmotic solution (Figure 2a), the a* colour parameter, describing red colour, was unchanged, and the L* and b* parameter mainly changed. Thus, the b* parameter, describing yellow colour, was lower after OD process, and the lowest value was obtained for samples dehydrated in mannitol solution.

Taking into account all the colour parameters, on the basis of ΔE, it can be concluded that the colour of the strawberry tissue after the OD process differed noticeably from fresh strawberry. In addition, Macedo et al. [48] found colour changes in dried strawberries subjected to prior drying and osmotic dehydration in 35% isomaltulose solution in comparison to fresh samples. The greatest changes in total colour difference were noted for the tissues subjected to OD in mannitol (ΔE > 10.5), which might be related to the creation of crystal forms in the tissue (see Section 2.5). Osmotic dehydration in the sucrose solution also resulted in large colour changes (ΔE = 10.8), which was comparable to the strawberries dehydrated in the mannitol solution. Conversely, sorbitol, as an osmotic substance, had the slightest effect on the colour of strawberries. However, as shown by de Oliveira et al. [49], the colour of the osmotically dehydrated yacon in reuse sorbitol solution under pulsed vacuum OD changed significantly with the increasing cycles of use of the solution.

### 2.3. Effect of Different Concentrations of Polyols on Bioactive Compound Contents in Organic Strawberries

#### 2.3.1. Total Anthocyanin Content (TAC) in Organic Strawberries Subjected to Osmotic Dehydration in Sucrose and Polyol Solutions

The total anthocyanin content (TAC) in organic strawberries, fresh and subjected to 3 h of osmotic dehydration (OD) in sucrose and polyols (mannitol, sorbitol) solutions, are presented in Figure 2a. In comparison to fresh organic strawberries (29.7 ± 0.2 mg Cyd-3-glu/g d.m.), the OD process contributed to a significant decrease in TAC, which was in the range of 49–67% (9.8–15.1 mg Cyd-3-glu/g d.m.). As Luo et al. [38] reported, the anthocyanins are mostly present in the cell’s vacuole. Therefore, during osmotic dehydration, they may leak from the cell into an osmotic solution. The same observations were made by Nowacka et al. [40] in the case of total anthocyanin content in cranberries subjected to different treatments before OD in sucrose or in sucrose with steviol glycosides. In addition, Macedo et al. [48] reported an analogous decrease in anthocyanin content in dried strawberries after osmotic pretreatment in isomaltulose solution in comparison to dried strawberries without osmotic treatment.

When analysing the osmotic agent, there was no difference in TAC in osmo-dehydrated strawberries between sucrose and sorbitol as well as sucrose and mannitol solutions. In the case of mannitol, the concentration of the osmotic substance did not significantly affect the content of anthocyanins. In turn, the utilization of sorbitol solution of a concentration of 30% and 40% resulted in a statistically greater loss of anthocyanins than for the concentration of 20%. However, statistically irrelevant changes between strawberries OD in sorbitol (20–40%) and in sucrose were noted. Comparing these results with the colour analysis and OD kinetics, it seems that the leakage of anthocyanins during osmotic dehydration was not the only reason for lower content of anthocyanins and colour change. Statistically irrelevant differences in a* parameter (describing red colour) was observed between fresh and osmo-dehydrated strawberries, but the degradation of anthocyanins after OD was noted. Among osmo-dehydrated materials, statistically, the highest TAC was obtained in strawberries OD in 20% solution of sorbitol. At the same time, for this concentration of sorbitol, the water loss and the total mass loss during OD were the lowest and statistically the same as in the case of mannitol 20%, for which statistically, the lowest TAC was determined. Perhaps the osmotic reagent also plays a role in greater or lower leakage or degradation of anthocyanins during OD, but the influence of a type of osmotic solution and its concentration was ambiguous and not clear. A deeper analysis of anthocyanins profile after OD in different solutions is needed.

#### 2.3.2. Total Phenolics Content in Organic Strawberries Subjected to Osmotic Dehydration in Sucrose and Polyol Solutions

In comparison to fresh strawberry slices (19.2 ± 0.9 mg chlorocenic acid/g d.m.) after osmotic dehydration, a decrease in TPC was noted (Figure 2b), but the changes were statistically relevant only when sorbitol solution of a concentration of 30% and 40% (15.8 ± 0.7 and 15.0 ± 0.1 mg chlorocenic acid/g d.m., respectively) were used as an osmotic substance. Perhaps the decrease in TPC in OD strawberries resulted from a decrease in TAC. OD of strawberries in sucrose, mannitol (20% and 30%) and sorbitol (20%) contributed to an unchanged content of polyphenols (16.0 ± 1.3, 17.1 ± 0.9, 16.3 ± 0.9, 17.3 ± 0.1, mg chlorocenic acid/g d.m., respectively), which indicates a protective effect of sucrose and mannitol solutions on the polyphenols in organic strawberries. These conclusions were in line with the results of TAC. Furthermore, there were no significant differences between different concentrations of the same variant of osmotic reagent. Thus, it seems that the type of osmotic substance was of greater importance than its concentration. Amami et al. [50] investigated the influence of ultrasound-assisted osmotic dehydration of strawberries in sucrose solutions before air drying. They reported a decrease in TPC in dried material subjected to OD, but it was almost the same as in the case of dried material without OD. Sakooei-Vayghan et al. [51] reported a significant decrease in TPC in apricot after OD in sorbitol solution for 30 and 45 min, compared with the fresh sample. However, after ultrasound-assisted OD for the same time, the losses were significantly greater than in apricot OD without sonication.

#### 2.3.3. Vitamin C Content in Organic Strawberries Subjected to Osmotic Dehydration in Sucrose and Polyol Solutions

The OD process caused a significant decrease in vitamin C regardless of the type of the osmotic solution. After OD, the content of vitamin C ranged between 22% and 43% of the vitamin C content in the fresh fruit (Figure 2c). In fresh strawberries, the amount of vitamin C was equal to 1.79 ± 0.04 mg/g d.m., while in the osmotic dehydrated material, the amount decreased to 0.39–0.77 mg/g d.m.

Considering the concentration of the osmotic substance, the amount of vitamin C decreased with an increase in the amount of the osmotic substance in the solution. These results, together with the results obtained from the analysis of OD kinetics, suggest that the vitamin C losses during OD may be explained by its leakage from the fruit to the solution as a result of the osmotic pressure gradient. However, the transfer of vitamin C has a negligible impact on mass exchange during OD, and it may be affected by many factors. This statement may be confirmed by studying the influence of type of osmotic agent on vitamin C content in strawberry, which turned out to be not clear. When comparing 20% mannitol and sorbitol solutions, it can be seen that the vitamin C content is significantly higher in the case of the use of mannitol versus sorbitol, but the samples osmo-dehydrated in 30% sorbitol solution were characterized by considerably higher amounts of vitamin C versus the samples osmo-dehydrated in 30% mannitol solution. Additionally, samples osmo-dehydrated in mannitol and sorbitol solutions at 20% and 30% concentrations showed the same relationships concerning vitamin C and polyol content. When analysing samples dehydrated in 20% mannitol and sorbitol solutions, mannitol gain is higher than sorbitol gain, while gains of mannitol and sorbitol in samples osmo-dehydrated in 30% solutions reveal the opposite tendency. These results may be explained by different impacts of the tested substances at different concentrations on the structure of the samples (see Section 2.5). Sorbitol and mannitol have different properties, they vary especially in solubility and crystals formation, and they thus influence differently on vitamin C content in osmo-dehydrated strawberries.

OD in 50% sucrose solution caused a reduction in vitamin C content to the level that did not differ statistically from the level of vitamin C caused by OD in 30% sorbitol solution and was significantly higher than the level of vitamin C in the sample osmo-dehydrated in 40% sorbitol solution. However, Hui et al. [52] observed that OD of pineapple in solutions at 65% (*w*/*w*) sucrose level caused higher loss of vitamin C when sucrose was used as an osmotic agent versus in the case of the use of sorbitol. In both cases, a considerable vitamin C loss was observed, and it amounted to 75.59% and 58.16% for sucrose and sorbitol solutions, respectively. Researchers also investigated the impact of duration of storage on vitamin C content. After 11 weeks of storage of osmo-dehydrated pineapple, vitamin C loss equalled 97.05% and 97.47% for samples dehydrated in sucrose and sorbitol solutions, respectively.

#### 2.3.4. Antioxidant Activity of Organic Strawberries Subjected to Osmotic Dehydration in Sucrose and Polyol Solutions

**Figure 3:**
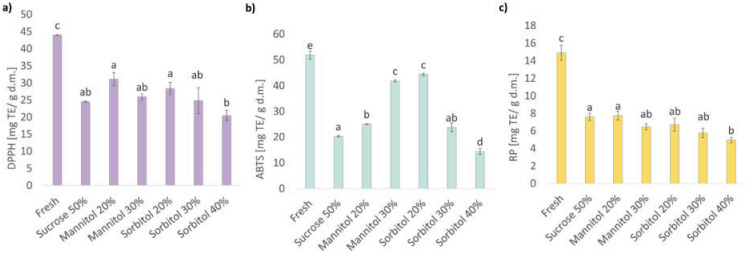
Antioxidant activity of organic strawberries after 3 h of osmotic dehydration in sucrose, mannitol, and sorbitol solutions, assessed with: (**a**) DPPH (1,1-diphenyl-2-picrylhydrazyl radical); (**b**) ABTS (2,2-azinobis (3-ethylbenzothiazoline-6-sulfonate) radical). (**c**) RP—reducing power, a–e—different letters above columns indicate statistically significant differences between the samples by the Tukey test (*p* < 0.05).

Osmotic dehydration reduced antioxidant activity of all investigated samples by 29–53%, 14–72%, and 48–67% when compared to the fresh material for DPPH, ABTS and RP assays, respectively (Figure 3). Tylewicz et al. [5] reported no significant changes or decreases in free radical scavenging activity of OD strawberries for DPPH and ORAC assay, respectively. In turn, Kowalska et al. [53] reported that OD in sucrose can improve the antioxidant activity of strawberries, which clearly shows that variety and origin of fruit are of paramount importance. Such results correspond well with the reported anthocyanins or vitamin C content, which belong to the main antioxidants of strawberries [54]. Despite that all techniques that have been used to evaluate the antioxidant activity are based on electron transfer (ET) [55], the biggest differentiation in the antioxidant activity was found using ABTS assay. In the case of this method, investigated variants were grouped into five homogenous groups, whereas in the case of DPPH and RP protocols, only three groups were determined. Moreover, the differences within dehydrated samples measured by DPPH and RP were also the smallest. For instance, the DPPH assay showed a significant difference only between fruits dehydrated in 20% mannitol, 20% sorbitol, and 40% sorbitol. In turn, RP protocol differentiated only samples obtained by OD in 50% sucrose, 20% mannitol, and 40% sorbitol. The best performance of the ABTS assay can be associated with the optical properties of the strawberry extract and the reaction mixture. From all utilized assays, DPPH and RP significantly correlated with TAC (DPPH: r = 0.915, RP: r = 0.951), TPC (DPPH: r = 0.973, RP: r = 0.903) and vitamin C (DPPH: r = 0.966, RP: r = 0.076). In turn, ABTS significantly correlated only with TAC and TPC (r = 0.773 and r = 0.833, respectively), whereas the relationship with vitamin C content was insignificant (r = 0.626).

Fruits dehydrated in 40% sorbitol solution exhibited the lowest antioxidant properties regardless of the measurement assay. Nevertheless, deeper analysis of the antioxidant activity measured by ABTS assay does not allow for one to make unambiguous conclusions when it comes to the influence of osmotic agent or its concentration. The samples dehydrated in polyols at concentrations below 40% exhibited better antioxidant activity (as measured by ABTS assay) than strawberries processed in traditional sucrose solution. Such a situation can be explained by the fact that mannitol and sorbitol exhibit protective properties against osmotic stress, although in such high concentrations, they can act as an osmotic agent [56,57]. The increment of concertation increased the antioxidant activity in the case of mannitol but decreased it linearly in the case of sorbitol. Considering that both sorbitol and mannitol exhibit similar radical scavenging activity [58], the results may be related to some other factors such as plasticizing of the cell’s biopolymers which may affect the mobility of water and water-soluble compounds [59,60]. Furthermore, Pearson’s correlation showed strong negative correlation of total mass loss (M_t_°) and water mass loss (M_t_^w^) with all bioactive compounds as the TAC (M_t_°: r = −0.959, M_t_^w^: r = −0.953), TPC (M_t_°: r = −0.910, M_t_^w^: r = −0.923) and vitamin C (M_t_°: r = −0.976, M_t_^w^: r = −0.974). In addition, this correlation was noted for antioxidant activity assessed with DPPH (M_t_°: r = −0.962, M_t_^w^: r = −0.970) and RP (M_t_°: r = −0.947, M_t_^w^: r = −0.941), whereas the relationship with ABTS was insignificant.

### 2.4. Effect of Different Concentrations of Polyols on Sugars and Polyol Content in Organic Strawberries

**Figure 4:**
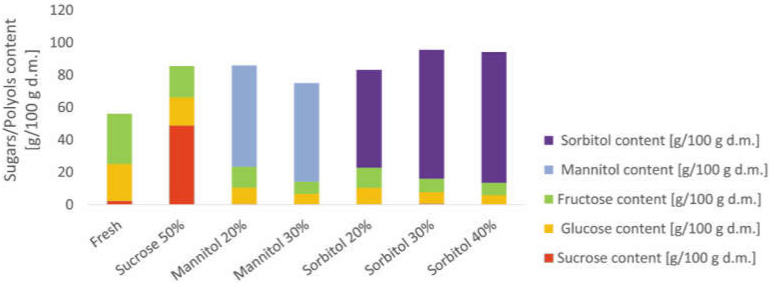
Sugar and polyol contents in organic strawberries after 3 h of osmotic dehydration in sucrose, mannitol, and sorbitol solutions.

The main sugars in strawberries are monosaccharide glucose, fructose, and disaccharide sucrose [61]. A similar sugar profile was obtained for organic strawberries, which are characterized by fructose of 31.04 ± 0.26 g/100g d.m., glucose of 22.70 ± 0.80 g/100g d.m. and sucrose of 2.39 ± 0.07 g/100 g d.m. (Figure 4). It is well known that the variety [22], as well as the method of cultivation (traditional and organic) [61], have an impact on the composition of fruit.

As expected, the osmotic dehydration process as well as the type of osmotic substances used in the process resulted in significant changes in the strawberries’ sugars profile (Figure 4). The changes in the sugars profile are the result of mass transfer and increases in the duration of the process, as demonstrated by Kowalska et al. [31] for osmo-dehydrated apples in sucrose, erythritol, xylitol, and maltitol solutions. When the osmotic dehydration process was conducted in sucrose solution, the strawberry tissue contained more sucrose, and its value increased from 2.39 ± 0.07 to 48.85 ± 1.36 g/100 g d.m. Similar observations were conducted by Cichowska et al. [22] in apple tissue dehydrated in sucrose and polyols as erythritol, xylitol and maltitol. In our study, the glucose and fructose content in strawberries sample-treated with sucrose solution were reduced by about 23.8% and 37.5%, respectively. These changes show that sugars from the tissue moved to the osmotic solution.

In the case of polyols, the sucrose was at a low level in the range of 0.12 to 0.62 g/100 g d.m., which did not differentiate samples (*p* > 0.05). In this case, glucose and fructose were also reduced by about 55.5% to 76%, depending on the used concentration and type of polyol. Statistical analysis showed that strawberries subjected to osmotic dehydration in polyols with lower concentrations (20%) contained a higher amount of glucose and fructose compared to solutions with a higher concentration (30% and 40%). On this basis, sugars in plant tissue were partially replaced by polyol content. Furthermore, when solutions with a higher concentration were used, larger amounts of sugars in tissue were replaced by polyols.

In order to use polyols on an industrial scale for the osmotic dehydration process, it is necessary to test the polyol content in the final product. This necessity is related to the possible laxative effect following the excessive consumption of polyols [27]. According to Wang and Eys [62], mannitol has a greater laxative effect. Furthermore, Cichowska-Bogusz et al. [30] showed that apples after the osmotic dehydration process and drying that were considered as a snack, did not contain a high amount of polyol residue, which can cause gastric problems.

### 2.5. Effect of Different Concentrations of Polyols on Organic Strawberry Structure

**Figure 5:**
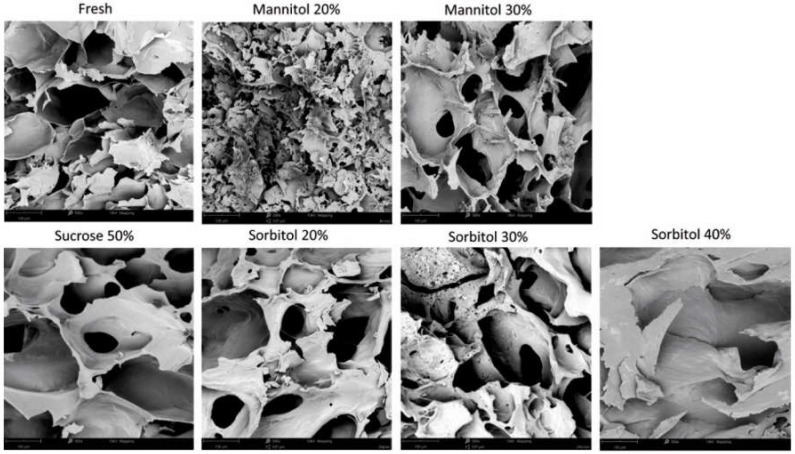
Microstructural changes (magnification 500×) of organic strawberries after 3 h of osmotic dehydration in sucrose, mannitol, and sorbitol solutions.

Due to the bidirectional mass exchange during the osmotic dehydration process, the internal structure of the strawberry tissue undergoes alterations [7,8,9]. Figure 5 shows the microstructure of fresh organic strawberries after 3 h of OD conducted in sucrose, mannitol, and sorbitol solutions. Intact strawberry tissue was characterized by cells with a round shape. After 3 h of osmotic dehydration in 50% sucrose solution resulted in numerous breakdowns of cell walls, the cells became irregularly shaped, distorted, and the formation of a non-uniform structure occur. These effects were also noted in the polyol solution. The use of a higher concentration of polyols resulted in greater structure changes. Increasing the concentration results in an increase in water loss and an increase in dry matter mass during osmotic dehydration (Figure 1) due to the fact that the difference in osmotic pressure between the hypertonic solution and the plant material increases [63].

**Figure 6:**
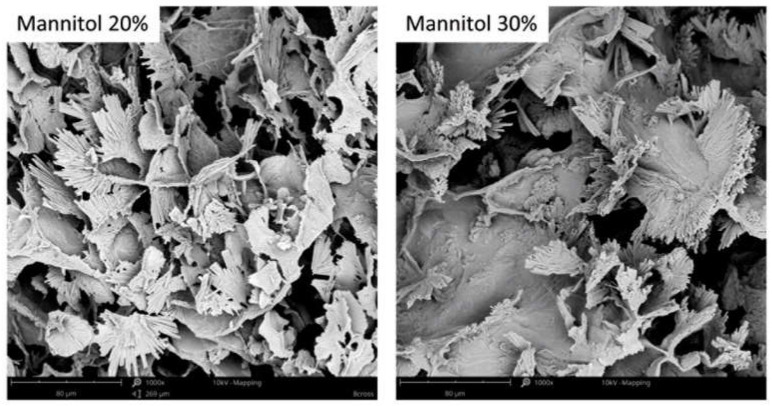
Mannitol crystals in organic strawberries after 3 h of osmotic dehydration (magnification 1000×).

Furthermore, for osmotic dehydration in mannitol solution, which is less soluble than sorbitol [36], the formation of mannitol crystals in the strawberry structure was observed (Figure 6). The concentration of the osmotic substance influences the increase in dry substance mass (Table 1); therefore, more polyols can be expected in the strawberry tissue when a higher concentration of the solution was used. This effect can be observed after 3 h of osmotic dehydration, in which the mannitol crystals were quite richly embedded in the strawberry tissue.

### 2.6. Cluster Analysis (CA)

**Figure 7:**
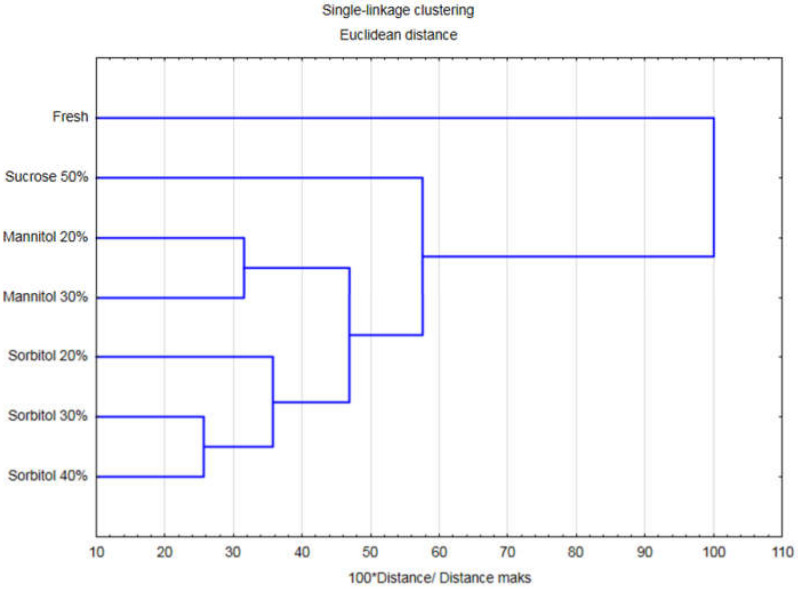
Cluster analysis of organic strawberries after 3 h of osmotic dehydration in sucrose, mannitol, and sorbitol solutions.

Cluster analysis was performed considering all of the evaluated process kinetics and product quality parameters. Thus, such an approach allowed for a complex comparison of all investigated technological variants. Agglomeration distinguished two major groups of the samples: fresh strawberries and dehydrated fruits (Figure 7). Within dehydrated fruits, three smaller clusters were determined. Despite similarities in some of the analysed properties, for instance TAC, TPC or antioxidant activity, when all parameters were considered, experimental variants were grouped according to utilized osmotic agents. Hence, strawberries dehydrated in sorbitol formed one cluster, regardless of the concentration. Similar results were found for mannitol and sucrose. Utilization of 30% and 40% solutions of sorbitol resulted in samples characterized by a high level of similarity. Such results may indicate that there is no need to utilize higher concentration when it comes to outcomes of the process, which should positively impact processing costs.

## 3. Materials and Methods

### 3.1. Materials

The research material was strawberries of the Roxana variety from certified organic farming. Fresh fruit was purchased at a farm located in Magnuszewo (Masovian Voivodeship, Poland) in one batch on the test day and was stored under refrigeration before use. Before the experiment, the strawberries were left at room temperature until they reached equilibrium. The fruits were washed in tap water and cut into 5 mm thick slices parallel to the calyx with a strawberry cutter.

### 3.2. Experimental Design

**Figure 8:**
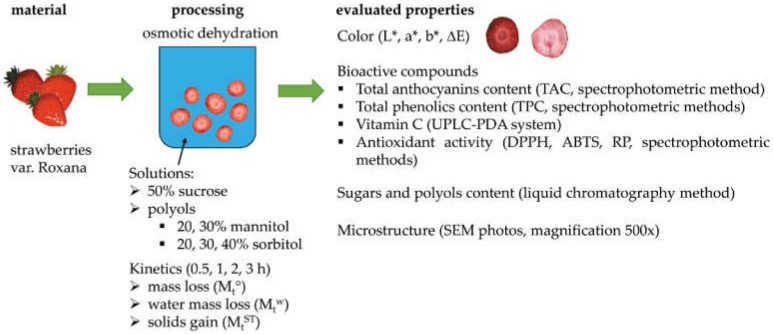
Experiment design for organic strawberries subjected to osmotic dehydration in sucrose, mannitol, and sorbitol solutions.

The samples were subjected to the procedures presented in Figure 8. The solutions for osmotic dehydration were prepared by dissolving the appropriate amount of sucrose, mannitol and sorbitol (from a local market) in distilled water heated to 50 °C, obtaining mixtures with concentrations of 50% for sucrose, 20% and 30% for mannitol and 20%, 30%, 40% for sorbitol. There was also a 40% mannitol solution prepared, but due to its low solubility, crystallization of the mannitol took place in the solution. Thus, this solution was not further studied. The characterizations the osmotic solution, the molar mass of the substances, and osmolality are presented in Table 3.

### 3.3. Osmotic Dehydration Process in Sucrose and Polyols

The osmotic dehydration process was carried out in glass vessels by completely immersing the cut material in solutions in a mass ratio of 1:4. A 50% sucrose solution was used as a reference solution. Osmotic dehydration was performed at 30 ° C for 3 h in a VSLB18 shaking water bath (VWR International, Radnor, PA, USA). The rotational speed of 100 rpm with an amplitude equal to 4 was used. After the times of 0.5, 1, 2, and 3 h, the samples were separated from the solution on a metal sieve and dried for 10 s on both sides on filter paper. For strawberries, the kinetics of the dehydration process was determined by calculating the total mass loss (M_t_°), water mass (M_t_^w^) loss and solids gain (M_t_^ST^) [65]. The osmotic dehydration process was carried out in duplicate.

Fresh and osmotically dehydrated strawberries for chemical analyses and internal structure were preserved using the freeze-drying process [66]. Fresh and osmotic dehydrated strawberries were shock-frozen (Shock Freezer HCM 51.20, Irinox, Treviso, Italy) at −40 °C for 4 h. Then, the material was freeze-dried in a laboratory freeze dryer (Gamma 1–16 LSC, Martin Christ Gefriertrocknungsanlagen GmbH, Osterode am Harz, Germany) with the following parameters: shelf temperature: 40 °C, pressure: 0.630 mbar, condenser temperature: −55 °C, drying time: 48 h. After drying, the material was stored in air and light barrier PET/AL/PE bags (Pakmar, Warsaw, Poland).

### 3.4. Physical and Chemical Properties of Organic Strawberries Subjected to Osmotic Dehydration in Sucrose and Polyol Solutions

#### 3.4.1. Dry Matter Content in Organic Strawberries Subjected to Osmotic Dehydration

The dry matter content in fresh and dehydrated material was determined by the gravimetric method by drying the ground samples at 70 °C for 24 h according to AOAC, 2002 [67]. The determination was performed in four replications.

#### 3.4.2. Colour Parameters of Organic Strawberries Subjected to Osmotic Dehydration

The colour measurement was carried out by the reflection method using a Konica Minolta CR-5 colourimeter (Konica Minolta Bench-top, Japan). Component values were recorded in the CIE L*a*b* system, where L* colour brightness ranges from 0 (black) to 100 (white), the a* parameter characterizes green (negative values) and red (positive values), while the b* parameter characterizes blue (negative values) and yellow (positive values). Standard light source D65, standard observer 2°, and a diameter of 3 mm of measuring gap were used. The actual tests were preceded by calibration with the use of a white and black standard. The measurement was performed in at least 10 replicates for each treatment. Based on the results, the total colour difference ΔE, according to the following equation [68]:

(1) $$\Delta E=\sqrt{{\Delta L}^{*2}+{\Delta a}^{*2}+{\Delta b}^{*2}},$$

where ΔL*, Δa*, Δb* are the differences of mean L*, a*, and b* parameters, respectively, between fresh strawberries and treated samples.

#### 3.4.3. Bioactive Compounds in Organic Strawberries Subjected to Osmotic Dehydration

##### Extraction Procedure

The same extraction procedure was carried out to analyse the content of anthocyanin, polyphenol, and antioxidant properties according to Nowacka et al. [69] with slight modifications. The dried material was ground in an analytical mill (IKA A11 basic; IKA-Werke GmbH, Staufen, Germany). For a falcon with a capacity of 15 mL, about 0.3 g of the material and 10 mL of the extraction reagent (80% ethyl alcohol + 0.1 M hydrochloric acid in the ratio 85:15) was added. Anthocyanin is more stable under acid conditions [70]. Extraction was performed on a shaker (Multi Reax, Heidolph Instruments, Schwabach, Germany) for 12 h at room temperature and with limited access to light. The solution was centrifuged for 2 min at 3000 rpm in a laboratory centrifuge (MegaStar 600, VWR, Leuven, Belgium). The supernatant was placed in 0.2 mL PCR tubes. Two extractions were made for each sample.

##### Total Anthocyanin Content of Organic Strawberries Subjected to Osmotic Dehydration

The quantitative determination of the total content of monomeric anthocyanin was carried out using the differential pH method [71]. For the analysis, buffers with pH 1 (1.86 g KCl in 1 L of water adjusted to pH with concentrated HCl) and pH 4.5 (54.43 g CH_3_CO_2_Na · 3H_2_O in 1 L of water adjusted to pH 4.5 with concentrated HCl) were used. Reactions were run on a 96-well plate. Then, 30 µL of extract solution and 135 µL of buffer were pipetted into the well and mixed. After 20 min incubation at 25 °C, the absorbance of the solutions was measured at 510 and 700 nm using a plate reader (Multiskan Sky, Thermo Electron Co., Waltham, MA, USA). At the same time, a reagent sample containing 30 µL of extraction reagent and 135 µL of buffer was measured. The determination was carried out in duplicate for each extract.

The total anthocyanin content (TAC) was calculated on the basis of the equation:

TAC [mg Cyd-3-glu/kg] = (A · MW · DF · 1000)/(ϵ · L), (2)

where:

A = (A_510_–A_700_) _pH1_ − (A_510_–A_700_) _pH4.5_;
MW—molecular weight of cyanide 3-glucoside (449.2 g/mol);
DF—sample dilution factor;
ϵ—molar absorption coefficient of cyanidin-3-glucoside (26 900 L/mol×cm);
L—optical path length for the solution in the well (0.173 cm).

##### Total Phenolics Content in Organic Strawberries Subjected to Osmotic Dehydration

The total content of polyphenolic compounds was determined by spectrophotometric method using the Folin–Ciocalteu reagent according to Singleton and Rossi’s method [72]. First, 10 µL of ethanolic extract and 10 µL of distilled water were dispensed into 96-well plates. Then, 40 µL of 5-fold diluted Folin’s reagent was added, mixed, and then, after 3 min of reaction, 250 µL of 7% sodium carbonate solution was insert. Incubations were performed for 60 min in a dark at room temperature. The absorbance was measured using a plate reader (Multiskan Sky, Thermo Electron Co., Waltham, MA, USA) at a wavelength of 750 nm. A blank test was also prepared in the same way, and the extract was replaced with an extraction reagent. The analysis was performed in duplicate for each extract. The quantitative of total polyphenol content was presented in relation to the calibration curve made for chlorogenic acid in the range of 0–100 µg/mL. The results were expressed as mg of chlorogenic acid per 1 g of dry substance of samples.

##### Vitamin C Content in Organic Strawberries Subjected to Osmotic Dehydration

Measurement of the amount of L (-) ascorbic acid was performed using a UPLC-PDA system [71]. To avoid vitamin loss, extraction was performed immediately prior to analysis. First, 0.2 g of grinded material was weighed and mixed with 10 mL of cooled extraction reagent (3% metaphosphoric acid, 8% acetic acid), 10 min vortexed and then centrifuged (5 min, 6000 rpm, 4 °C). All activities were carried out with limited access to light. The supernatant was filtered through 0.22 µm polypropylene syringe filters (GHP Acrodisc, Pall Gelman, Ann Arbor, MI, USA). Then, 1 mL of solution was added to 1 mL of eluent (Milli-Q water with 0.1% formic acid) and was injected onto the column (5 mL). Separation was performed on a WATERS Acquity UPLC HSS T3 chromatography column (2.1 × 100 mm, 1.8 µm; Waters, Ireland). The mobile phase (eluent) flow was 0.25 mL/min, the temperature of the column thermostat was 25 °C, and the samples were kept at 4 °C. The spectrum was analysed at 245 nm. The content of vitamin C in the samples was determined on the basis of the calibration curve prepared for the standard of L(-) ascorbic acid in the range of 0.005–0.1 mg/mL. The analysis was performed in duplicate.

##### Antioxidant Activity of Organic Strawberries Subjected to Osmotic Dehydration

To assess the antioxidant properties of samples, spectrophotometric methods were used, which consisted of determining the ability to reduce Fe^3+^ ions (RP), the 2,2-diphenyl-1-picrylhydrazyl radical (DPPH) and the cation radical 2,2-azinobis (3-ethylbenzothiazoline-6-sulfonate) (ABTS).

##### Determination of the Antioxidant Capacity against DPPH and ABTS Radicals

In order to generate free radicals, stock solutions of DPPH and ABTS were prepared 24 h before the analysis [55]. First, 25 mg of 2,2-diphenyl-1-picrylhydrazyl was weighed into a 100 mL volumetric flask and made up to 100 mL with a 99% methanol solution. The ABTS solution was prepared by dissolving 38.4 mg of 2,2-azinobis (3-ethylbenzothiazoline-6-sulfonate) in 10 mL of distilled water and adding 6.6 mg of potassium persulfate. The solutions were stored in the refrigerator. Before the analysis, working solutions of the radicals were prepared by diluting the stock solutions with 80% ethanol to obtain a concentration showing absorption in a 1 cm cuvette at a wavelength of 515 nm for DPPH and 734 nm for ABTS, at about 0.7 AU (absorbance unit).

Reactions were performed in 96-well plates. The analyte solution was diluted 5 times. First, 10 µL of extract and 250 µL of radical solution were added to the well, mixed and measure the absorbance for DPPH after 10 min at 515 nm, for ABTS after 6 min at 734 nm versus 80% ethanol. At the same time, the absorbance of the radical working solutions was checked. The antiradical activity was determined from the decrease in the absorbance of the radical solution in the presence of an antioxidant and expressed as mg Trolox/g dried material. The determination was carried out in duplicate for each extract.

##### Reducing Power (RP)

The analysis was performed based on the methodology described by Świeca [73], with slight modifications. In a 96 well plate, 25 µL of the extract, 75 µL of distilled water and 50 µL of 1% aqueous potassium ferric cyanide solution were mixed. The mixture was placed in the dark at 50 °C in an incubator (INCU-Line ILS 10; VWR, Radnor, PA, USA). After 20 min, 50 µL of 10% trichloroacetic acid was added. Then, 100 µL of the solution was taken into the empty well and 100 µL of distilled water, 20 µL of 0.1% iron (III) chloride solution were added and mixed. After 10 min, the absorbance values of the solutions were measured at 700 nm against the reagent sample using a plate reader. The value of the iron ion reduction force for each sample was expressed as mg of Trolox. The determination was carried out in duplicate.

#### 3.4.4. Sugar and Polyol Content in Organic Strawberries Subjected to Osmotic Dehydration

The method of liquid chromatography with the detection of the refractive index was used to determine the sugar and polyol content [31]. The system was equipped with a quadruple pump (Waters 515, Milford, MA, USA), autosampler (Waters 717, Milford, MA, USA), column thermostat and RI detector (Waters 2414, Milford, MA, USA). Separation was carried out using a 300 × 6.5 mm Waters Sugar Pak I column with a Sugar-Pak precolumn. Then, 0.2 g of ground material was extracted with 10 mL of ultra-pure water at 80 °C for 4 h. The solution was filtered through a 0.45 µm PTFE syringe filter and dispensed into the system. The injection volume was 1 µL. The analysis was performed under isocratic conditions, the flow rate of the mobile phase (Milli-Q ultra-pure water with 18.2 MΩ cm, temperature 25 °C) was 0.6 mL/min, the column temperature was 90 °C, and the detector temperature was 50 °C. Quantitative analysis was performed on the basis of prepared calibration curves for sucrose, glucose, fructose, mannitol and sorbitol standards. The determination was carried out in duplicate.

#### 3.4.5. Scanning Electron Microscopy Analysis (SEM)

The internal structure of the dried material was analysed using a Phenom XL scanning electron microscope (Phenom World, Eindhoven, the Netherlands). The dried material was screwed to a metal table and coated with a layer of gold (Cressington Sputter Coater 108 auto, Cressington Scientific Instruments, Watford, UK). The observations were made using a beam accelerating voltage of 10 kV and a vacuum at 60 Pa. Photos of the sample cross-section were recorded at a magnification of 500 and 1000.

### 3.5. Statistical Analysis

Statistical analysis was performed using Statistica 13 software (TIBCO Software, Palo Alto, CA, USA). Homogenous groups were determined using ANOVA and Tukey test (α = 0.05). In order to assess the relationship between selected variables, Pearson’s correlation analysis was performed. Moreover, the complex evaluation and differentiation of the samples based on all investigated parameters was performed using cluster analysis with single-linkage clustering and Euclidean distance.

## 4. Conclusions

The use of polyols such as mannitol and sorbitol affects the bioactive properties of organic osmotically dehydrated strawberries. The commonly used sucrose solution for the osmotic dehydration process can be replaced with polyol solutions. Sorbitol solution with a concentration of 30% can be recommended, within investigated parameters and variants, due to the close or similar course of the osmotic dehydration process of organic strawberries in sucrose solution. Furthermore, the use of the polyols resulted in unchanged bioactive substances for 30% sorbitol solution, which was lower for samples dehydrated in 40% sorbitol in comparison to sucrose solution. Similarly, the colour (ΔE) of samples dehydrated in polyols was comparable to strawberries dehydrated in sucrose solution. Generally, the results obtained for 30% and 40% sorbitol were similar to the sucrose solution; however, the profile of the sugar changed significantly. The sugars (sucrose, glucose, and fructose) in organic strawberries were partially replaced by the polyols. Due to slight differences between the obtained properties of dehydrated strawberries and the process carried out in 30% and 40% sorbitol solutions, as well as cost reduction, it is proposed to use a sorbitol solution with a concentration of 30%.

The use of a mannitol solution in the osmotic dehydration process is not recommended due to the lack of significant changes in comparison to sucrose solution in bioactive compounds and colour. Moreover, there are some difficulties with preparing highly concentrated solution, which is linked to its low solubility as well as crystallization of the mannitol in the tissue, which was confirmed by structural changes in scanning electron microscopy.

## Figures and Tables

**Table 1:** Total mass loss (M_t_°), water mass loss (M_t_^w^) and solids gain (M_t_^ST^) after 3 h of osmotic dehydration process of organic strawberries in sucrose, mannitol, and sorbitol solutions, a–c—different letters in columns indicate statistically significant differences between the samples by the Tukey test (*p* < 0.05).

Sample	ΔM_t_^o^ (kg/kg)	ΔM_t_^W^ (kg/kg)	ΔM_t_^ST^ (kg/kg)
Sucrose 50%	0.603 ± 0.020 b	0.657 ± 0.013 c	0.054 ± 0.006 c
Mannitol 20%	0.460 ± 0.037 a	0.477 ± 0.031 a	0.016 ± 0.006 ab
Mannitol 30%	0.523 ± 0.023 ab	0.564 ± 0.017 b	0.041 ± 0.005 bc
Sorbitol 20%	0.471 ± 0.008 a	0.486 ± 0.007 a	0.015 ± 0.001 a
Sorbitol 30%	0.538 ± 0.020 ab	0.578 ± 0.015 b	0.041 ± 0.005 bc
Sorbitol 40%	0.572 ± 0.032 b	0.627 ± 0.023 bc	0.055 ± 0.010 c

**Table 2:** Colour parameters (L*—lightness, a*—red colour, b*—yellow colour, ΔE—total colour difference calculated in comparison to fresh strawberries) of organic strawberries after 3 h of osmotic dehydration in sucrose, mannitol, and sorbitol solutions, a–c—different letters in columns indicate statistically significant differences between the samples by the Tukey test (*p* < 0.05).

Sample	L*	a*	b*	ΔE
Fresh	34.3 ± 2.4 a	25.8 ± 1.5 ab	21.4 ± 3.4 a	-
Sucrose 50%	43.6 ± 2.6 c	26.3 ± 3.0 a	17.9 ± 1.5 ab	10.8 ± 2.1 ab
Mannitol 20%	38.7 ± 1.7 b	22.3 ± 2.5 ab	12.9 ± 1.7 cd	10.5 ± 2.1 ab
Mannitol 30%	39.5 ± 2.9 b	22.0 ± 2.6 b	12.5 ± 2.6 d	11.5 ± 2.5 b
Sorbitol 20%	40.0 ± 2.7 bc	24.0 ± 2.0 ab	16.7 ± 2.3 bc	8.3 ± 2.0 a
Sorbitol 30%	40.9 ± 2.1 bc	24.9 ± 2.5 ab	16.3 ± 2.6 bcd	8.9 ± 2.7 ab
Sorbitol 40%	38.0 ± 1.3 ab	26.3 ± 2.7 a	15.0 ± 1.9 bcd	7.9 ± 1.8 a

**Table 3:** Characteristic of the osmotic solution.

Osmotic Solution	Molar Mass of Substance (g/mol)	Osmolality ^1^ (mOsm/kg H_2_O)
sucrose 50%	342.3	2921
mannitol 20%	182.2	1372
mannitol 30%	182.2	2353
sorbitol 20%	182.2	1372
sorbitol 30%	182.2	2353
sorbitol 40%	182.2	3660

^1^ Theoretical calculated osmolality based on Rasouli [64].

## Data Availability

The data presented in this study are available on request from the corresponding author.

## Sample Availability

Samples are not available.
